# Thrombotic complication during intracoronary imaging

**DOI:** 10.1007/s12471-012-0275-9

**Published:** 2012-04-27

**Authors:** S. A. Wiyono, H. M. M. van Beusekom, J. M. Ligthart, W. J. van der Giessen

**Affiliations:** 1 Thoraxcenter, Erasmus MC Rotterdam, ‘s Gravendijkwal 230, 3015 CE Rotterdam, the Netherlands; 2 Interuniversity Cardiology Institute of the Netherlands, ICIN-KNAW, Utrecht, the Netherlands; 3 Present Address: Department of Cardiology, University of Leuven, Leuven, Belgium

**Keywords:** Coronary stent, IVUS, OCT, Thrombosis

## Abstract

Intracoronary imaging with intracoronary ultrasound and coherence tomography is often used in the follow-up of coronary stent implantation. The present case shows an infrequent complication of these procedures, suggesting our continued attention to the selective use of these invasive procedures.

A 70-year-old male with a history of hypertension underwent follow-up coronary angiography and invasive imaging within the frame of a clinical study. Six months earlier he was admitted with a non-ST-elevation myocardial infarction with creatine kinase (CK) within the normal range and troponin T max 0.22 μg/l (normal ≤0.02). A significant stenosis at the bifurcation of the left anterior descending coronary artery (LAD) and first diagonal branch (LD1) was shown to be the culprit lesion, which was treated by implantation of a self-expanding, bare metal stent (Stentys coronary bifurcation system, Stentys SAS, Clichy, France). In addition, two everolimus-eluting stents (Xience-V, Abbott Vascular, St. Clara, CA) were placed in the distal LAD and diagonal branch to seal edge dissections as seen by intracoronary ultrasound (IVUS). No thrombus or tissue prolapse were demonstrated by IVUS or optical coherence tomography (OCT). The procedure was uneventful and the patient remained asymptomatic during follow-up.

The follow-up procedure was performed under double antiplatelet therapy and 7500 IU unfractionated heparin was administered 5 min before guiding catheter engagement in the left coronary artery. Angiography showed moderate diameter stenosis of a maximum of 36 % at the carina. IVUS catheter insertion (40 MHz Atlantis SR® Pro Imaging, Boston Scientific, Natick, MA) in the LAD proved difficult, but was successful after switching to a support guide wire. Imaging from the LD1 to the proximal LAD confirmed moderate in-stent neointimal hyperplasia without evidence of thrombus in the stent or the proximal LAD. Subsequent OCT imaging (Cx7, LightLab Imaging, Westfort, MA) demonstrated thrombus in the proximal LAD, but not in the stents. Repeated IVUS and OCT imaging, respectively, now also revealed thrombosis in the stents (Fig. 1a,b), but no sign of new dissections.

**Fig. 1:**
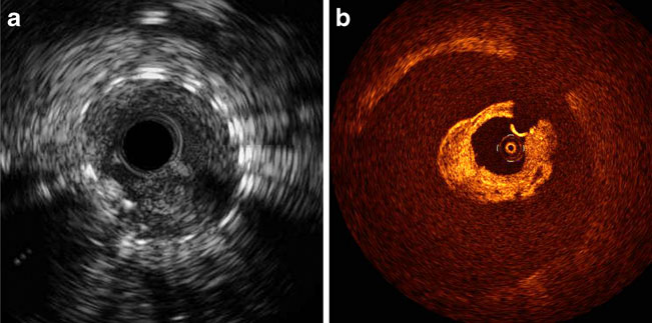
**a** IVUS imaging shows in-stent thrombosis in the bifurcation stent of the left anterior descending artery and first diagonal branch. **b** OCT imaging shows thrombus in the proximal LAD

Angiography immediately after IVUS and OCT imaging now also showed angiographic evidence of thrombus with subtotal occlusion of the proximal LAD, in-stent thrombosis, and blockage of the LD1 (Fig. 2a). Thrombus aspiration in both the LAD and LD1 was performed (Thrombuster, Kaneka Medics, Osaka, Japan) and yielded combined red- and white-coloured material. The activated clotting time (ACT) at this stage of the procedure was 515 s. Thrombus aspiration was followed by balloon dilatation of the stents in the LAD and the LD1. Intracoronary bolus and intravenous glycoprotein IIb/IIIa blockers were administered. The final angiogram showed recovery of TIMI 3 flow without visible thrombi (Fig. 2b). Post-procedure troponin T was slightly elevated (0.10 μg/l) without elevation of CK-MB.

**Fig. 2:**
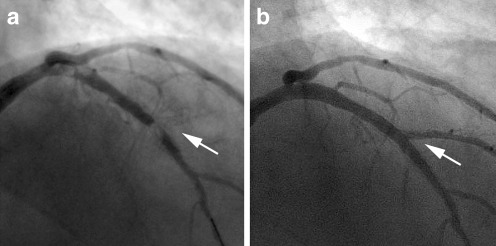
**a** Angiogram immediately after second IVUS and OCT pullbacks confirmed thromboembolism with subtotal occlusion of the proximal LAD, in-stent thrombosis in the LAD stent and blockage of the first diagonal branch (arrow). **b** Final angiogram following thrombus aspiration, balloon dilatation and administration of GP IIb/IIIa blockers shows recovery of normal flow without visible thrombi

Histology showed fresh thrombus composed of fields of erythrocytes surrounded by large amounts of platelets and fibrin. Collagen and neointimal tissue were not observed (Fig. 3).

**Fig. 3:**
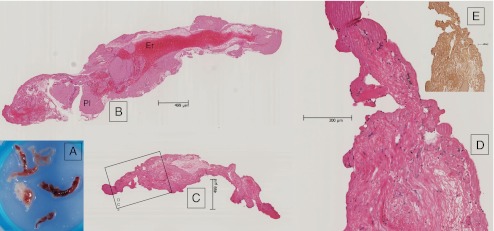
Macroscopy of the aspirate (**a**) shows both red and white thrombus. Histology (**b–e**) confirms the presence of fresh thrombus containing fields of erythrocytes (Er) and platelets (Pl). The resorcin-fuchsin stain in **e** (HE in **d**, detail of **c**) shows that the tissue does not contain collagen and indicates absence of neointima

## Discussion

Intracoronary imaging is regarded as useful for guidance of coronary interventions and evaluation of the response to (device) therapy. However, they are invasive procedures and may cause serious complications such as illustrated in the current case. Multicentre survey of 2207 IVUS examinations showed 2.9 % and 1 % of the cases suffered from coronary spasm and other complications, such as acute coronary occlusion, thromboembolism, dissection (with or without enzyme release) during IVUS imaging [1]. This is, however, rather dated data. Current IVUS systems are much lower profile than the catheters in the early 1990s.

Complications of OCT are less well studied, as this is a much younger technique. Guagliumi et al. reported one patient out of 141 with a non-Q-wave myocardial infarction with minimal increase of troponin I [2]. In multicentre evaluation of 468 patients, Gonzalo et al. reported no thromboembolism or major adverse cardiac events within 24 h following the OCT procedure [3].

## Conclusion

Intravascular imaging procedures such as IVUS and OCT are safe, but not free from major complications, even when procedural ACT is in the therapeutic range.
